# Supplementary material for: Incidence of Occult Hepatitis B Infection (OBI) and hepatitis B genotype characterization among blood donors in Cameroon
Source: PLoS One. 2024 Oct 16;19(10):e0312126. doi: 10.1371/journal.pone.0312126 (PMC11482724; doi:10.1371/journal.pone.0312126)
Supplement: S1 Table — (PDF) [file pone.0312126.s001.pdf]

Qualitative nested PCR	
HBV-022	5’-TGCTGCTATGCCTCATCTTC-3’
HBV-065	5’-CACAGATAACAAAAAATTGG-3’
HBV-066	5’-CAAAGACAAAAGAAAATTGG-3’
HBV-024	5’-CAAGGTATGTTGCCCGTTTGTCCT-3’
HBV-041	5’-GGACTCAMGATGYTGCACAG-3’
HBV-064	5’-GGACTCACGATGCTGTACAG-3’
**Quantitative qPCR**	
HBV-61	5’-GGACCCCTGCTCGTGTTACA
HBV-62	5’-GAGAGAAGTCCACCACGAGTCTAGA
HBV-TM-5	FAM 5'-TGTTGACAARAATCCTCACAATACCRCAGA-3' DabCyl
