# Supplementary figures and images for: Incidence of Occult Hepatitis B Infection (OBI) and hepatitis B genotype characterization among blood donors in Cameroon
Source: PLoS One. 2024 Oct 16;19(10):e0312126. doi: 10.1371/journal.pone.0312126 (PMC11482724; doi:10.1371/journal.pone.0312126)

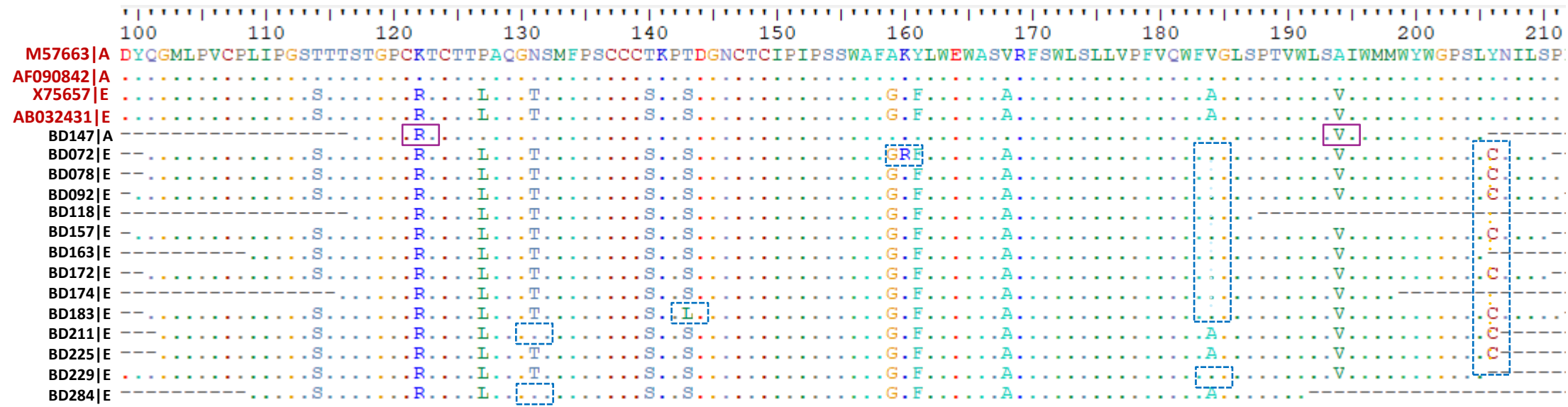

Supplement: S1 Fig — Surface protein alignment of OBI-positive sequences with reference sequences for genotypes A and E (reference genomes: M57663|A, AF090842|A, X75657|E, and AB032431|E written in red). The alignment shows amino acids 100 to 210 of the S protein. A dot represents homology in amino acids as observed in the reference sequence; only those with amino acid substitutions are illustrated. Bold squares indicate amino acid substitution for genotype A and dash lines show amino acid substitutions for genotype E. (PDF) [file pone.0312126.s002.pdf]

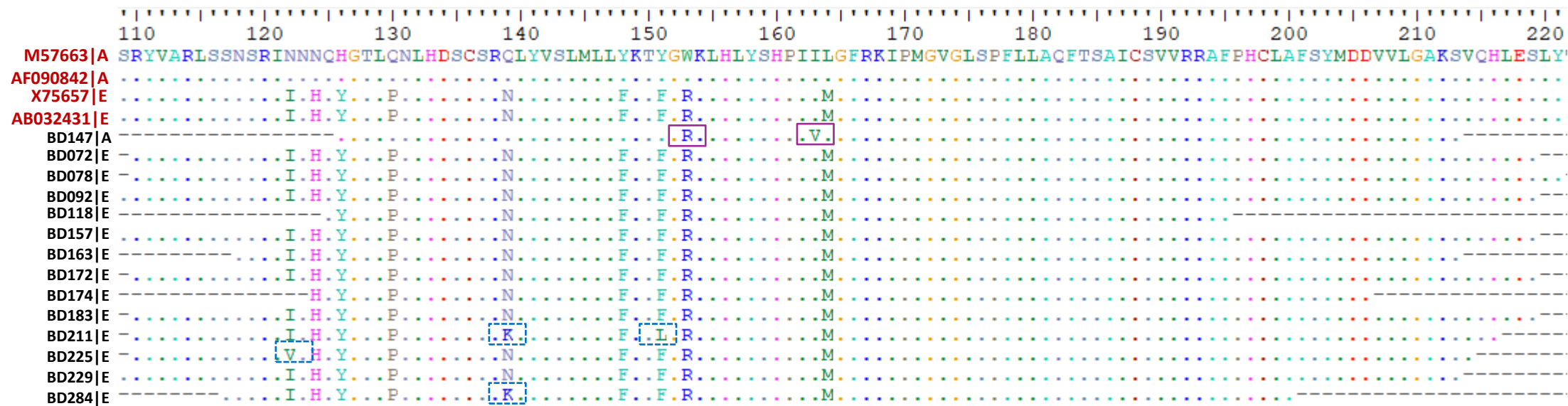

Supplement: S2 Fig — The alignment of the RT region of the P gene of OBI-positive sequences with reference sequences for genotypes A and E (reference genomes: M57663|A, AF090842|A, X75657|E, and AB032431|E, written in red). The alignment shows amino acids 110 to 220 of the RT domain. A dot represents homology in amino acids as observed in the reference sequence; only those with amino acid substitutions are illustrated. Bold squares indicate amino acid substitution for genotype A and dash lines show amino acid substitutions for genotype E. (PDF) [file pone.0312126.s003.pdf]
