# Supplementary material for: Incidence of Occult Hepatitis B Infection (OBI) and hepatitis B genotype characterization among blood donors in Cameroon
Source: PLoS One. 2024 Oct 16;19(10):e0312126. doi: 10.1371/journal.pone.0312126 (PMC11482724; doi:10.1371/journal.pone.0312126)
Supplement: S1 File — (PDF) [file pone.0312126.s004.pdf]

Information Sheet, Consent Form, and Questionnaire.

a) **Study title: Molecular surveillance of hepatitis E virus and occult hepatitis B infection in the Cameroonian Population.**

Name of researcher: **Mbencho Macqueen Ngum**

Institution: Institute of Tropical Medicine, Eberhard Karls University of Tuebingen, Germany

Information for participants

Thank you for considering participating in this study. This information sheet outlines the purpose of the study we want to do, and it describes your involvement and rights as a participant if you agree to take part.

1. What is the research about?

This study is about Hepatitis disease which affects the liver of humans. It is caused by a virus. There are many types of hepatitis such as Hepatitis A, B, C, D, and E. The focus of this study is on Hepatitis B and E diseases. One can have hepatitis B through blood transfusion and other body fluids, and also by contact with contaminated surfaces. Hepatitis E disease is gotten by drinking water and eating vegetables that have been contaminated with this virus, and also through blood transfusion. In addition, you could get this virus from eating pork (from pigs), rabbits, cow meat, and bushmeat. Also, you have a higher risk of falling sick from this virus if you are pregnant, you have other diseases like HIV, or you keep or sell animals, especially pigs. Therefore, we will like to find out how much of the population of South West Cameroon is suffering from these diseases.

2. Do I have to take part?

It is up to you to decide whether to take part. You do not have to take part if you do not want to. If you do decide to take part, I will ask you to sign a consent form which you will keep one copy and I will have one copy. You can participate only if you are between 18 and 60 years old.

3. What do I have to do in this study?

You will be asked to fill out a questionnaire, providing some information about yourself and your lifestyle which will guide us in the process of finding out whether you have this disease or not, and possibly how you got it if you have. Thereafter, a very small amount of blood and stool (excreta) will be collected from you. It will take just about 15 - 20 minutes of your time.

4. How do I withdraw from the study?

You can withdraw from the study at any point, with or without any reason. Even though I will appreciate it if you take part in every step of this study, you are free to stop the process at any time with no consequences on you whatsoever. And your information also will be deleted if you wish so.

5. What will my information or blood be used for?

The samples we will collect from you will be used only for the purpose of this study described in (1) above and nothing else. Also, only a small amount of your blood is needed so that there will be no wastage.

6. Will I feel any pain in the process?

Since blood will be collected with a needle, you might feel just very little pain. However, you don't need to be afraid because it will be done by a professional in the field to reduce your trauma to the minimum.

7. Will my information be kept confidential? Will you hide my identity?

The information provided by you in this study will be kept confidential. Only I alone will have access to them. Also, codes will be used on the samples instead of your names. And your coded questionnaires will be kept confidential too till when they will not be needed anymore.

8. What will I benefit from this study?

The only direct benefit you have from this study is that you will be informed whether you have hepatitis E virus disease or not. This will only be at the end of this study which might take several months. Also, there will be no financial compensation from me to you in this study.

9. Who has given you the right to carry out this study?

This study has undergone ethical review by the Institutional Review Board of the University of Buea. Authorization has been given by the Delegation of Public Health in the Southwest, and also by the Directors of this Hospital and unit.

10. What if I have a question or complaint?

If you have any further questions about this study, please feel free to ask me now, or contact me at **mcqueengum@gmail.com.** If you have any concerns or complaints regarding the conduct of this research, please contact my home supervisor Prof. Stephen Ghogomu at stephen.ghogomu@ubuea.cm

If you have read and understood this information sheet, all questions you had have been addressed, and you are happy to take part in this study, please sign the consent sheet below.

By signing this form, I hereby declare that;

I have read and understood the study information, or it has been explained to me. I have been able to ask questions about the study and my questions have been answered to my satisfaction.	YES / NO
I voluntarily give my consent to be a participant in this study and understand that I can refuse to answer questions and that I can withdraw from the study at any time, without having to give a reason.	YES / NO
I understand that the information I provide will be used for the doctoral thesis of the researcher, Mbencho Macqueen Ngum, and that the information will be anonymized.	YES / NO
I understand that any personal information that can identify me – such as my name, and address, will be kept confidential and anonymized when publishing the results from this study.	YES / NO

Participant name: …………………………………………………………………………………

Signature: ______________________________ Date ______________

Researcher's name:
………………………………………………………………………………………………………
….

Signature: ______________________________ Date ________________

Please keep a copy of this consent form!!!!!!!

QUESTIONNAIRE

Cohort__________________________________

Dear participant, kindly respond to the following questions by placing a tick (√) or a cross (X) in the box and filling in the blank spaces with the most appropriate response.

Section 1: General Information from all participants.

1. Participants' code ________________________________collection time_______________
2. Gender: Male ☐ Female ☐ Age ________
3. Marital Status: Single ☐ Married ☐ Divorced ☐ widow/widower ☐
4. Where do you stay? ____
5. What do you do (occupation)? ____
6. Level of Education: Primary school☐| Secondary school☐| High school☐| University level☐
7. How much do you earn in a month? Less than 40,000frs ☐ 40 – 100,000frs☐

   100 - 250,000frs☐ More than 300,000frs ☐
8. How many people live in your home? ________________
9. What type of water do you drink? Bottled water ☐ Tap water ☐ Spring/well water ☐

   Borehole water ☐
10. Do you eat pork? Yes ☐ No ☐

    Which other meat do you eat? ____
11. Do you eat Bushmeat? Yes ☐ No ☐
12. Do you buy vegetables from the market? Yes ☐ No ☐
13. How long do you usually cook or roast your meat? ________________________________
14. Do you sometimes taste or eat raw meat? Yes ☐ No ☐
15. Do you keep animals at home? Yes ☐ No ☐

    If yes, which animals? ____
16. Have you ever received blood in the hospital? Yes ☐ No ☐

    If yes, when last did you receive blood? ____
17. Have you ever donated blood? Yes ☐ No ☐ when last did you donate? ____________
18. Is this your first time hearing about the Hepatitis E virus? Yes☐ No ☐
19. Have you taken the hepatitis B vaccine? Yes No

    If yes, when did you take it?_______________________ How many doses? __________

Section B: For Pregnant women only.

20. How many months is your pregnancy? ____
21. Which other disease do you have? None ☐ Hepatitis B ☐ Hepatitis C ☐ HIV ☐

    Others____

22. Have you ever had a miscarriage? Yes ☐ No ☐ Ever delivered a dead baby? Yes ☐ No ☐

Section C: For HIV participants only.

23. What's your line of treatment? ____

24. How long have you had HIV? ____

25. Which other disease do you have? None ☐ Hepatitis B ☐ Hepatitis C ☐ HIV ☐

    Others____

Section D: For occupationally exposed only.

26. Which animals do you keep at home? Pigs ☐ Rabbits ☐ Goats ☐ others___________

    I do not keep animals ☐. How many animals do you have? __________________________

27. If you are a butcher or hunter, do you always touch the blood of the animals or meat with your bare hands? Yes ☐ No ☐

28. Telephone number ____

Participant's signature

____

THANK YOU **MERCI** **DANKE SCHÖN**

VALIDATION: YES………………. NO……………………...

(For researcher only)
