# Supplementary material for: Incidence of Occult Hepatitis B Infection (OBI) and hepatitis B genotype characterization among blood donors in Cameroon
Source: PLoS One. 2024 Oct 16;19(10):e0312126. doi: 10.1371/journal.pone.0312126 (PMC11482724; doi:10.1371/journal.pone.0312126)
Supplement: Attachment — Submitted filename: Rebuttals_OBI_PLOS_ONE_R1.docx [file pone.0312126.s005.docx]

**Editor comments:**

This is a cross-sectional study of occult HBV conducted in blood donors in Cameroon.Given the high burden of HBV in resource-limited settings, such studies are important. The population size is relatively modest and the finding of 14 occult HBV infections is not surprising.

We appreciate the editor's valuable comments. We acknowledge that the sample size in our study is modest, and identifying 14 cases of occult HBV infection, especially in Southwestern Cameroon, where the HBsAg positivity rate ranges from 8-15% (<https://pubmed.ncbi.nlm.nih.gov/28667212/>; reference 2 in the draft). The required sample size based on our power calculation was 225. Using available regional studies and our sample calculation (Sample size n = [DEFF*Np(1-p)]/ [(d2/Z21-α/2*(N-1)+p*(1-p)]), we estimated that investigating 225 blood donors (with 80% power and a 95% two-sided confidence interval) would allow us to detect the incidence in the general population. However, our current study included a larger sample size of 288 participants. This study is the first of its kind in this anglophone region (Buea) of Cameroon, where the majority of the country is francophone. Additionally, Northern Cameroon has faced years of insurgency and violence from Boko Haram, highlighting the significance of research in this region to assess the broader implications of occult hepatitis B infection among blood donors.

What region of the viral genome is targeted by the real-time PCR assay?  Were samples that tested negative for HBV DNA re-tested a second time?

Thank you for your insightful questions. The real-time PCR targeted the S gene of the HBV genome. We have now included this detail in the methods section as follows:

"*For DNA quantification, real-time PCR was conducted using virus-specific primers and a Taqman probe that targeted a 90-bp fragment within the conserved region of the S gene in the HBV genome (position 182-271, GenBank number X75657), following a previously described protocol [14]."*

Regarding your second question, All 288 samples were tested using both nested PCR and real-time PCR. Samples for HBV DNA were tested three times, and only those that tested positive at least twice by nested PCR were reported in this article.

By definition, occult HBV cases are HBsAg negative but HBV DNA positive.  Only 4 samples included in Table 1 actually have detectable HBV DNA by real-time PCR.

Yes, of the 14 OBI-positive cases, only 4 had detectable HBV viremia by quantitative PCR. All 14 samples were positive only by qualitative PCR, which was tested independently three times. Occult HBV infection (OBI) is characterized by the presence of low levels of HBV DNA in the serum, with a negative HBsAg test and often undetectable or very low viremia. In our study, we were able to detect viremia as low as 25 IU/mL only in 4 samples.

For the mutational analysis, what were the study sequences compared to?  Genotype-matched references?  How many? What was the definition of a mutation then?  Any mutation not detected in the reference set?  Above a certain threshold (say 10%)?

Based upon an inter-group genetic divergence of >8% in the complete nucleotide sequence, HBV are categorised into 10 genotypes (A to J). The HBV genotypes show different geographical distribution in populations around the globe. The most cosmopolitan genotypes are A and D, which are prevalent in Europe, Africa and North America. While Genotypes B and C are most common in East and Southeast Asia, and genotype E is mainly restricted to Africa. Genotype F is indigenous to the Americas and is found in native populations from Alaska, Central and South America.

In the context of hepatitis B virus genotyping, a mutation is defined as a change in the nucleotide sequence of the HBV genome compared to a reference sequence for a particular genotype. These mutations can result in changes at the amino acid level if they occur within coding regions of the virus, potentially altering the function or antigenicity of viral proteins. For our mutation analysis, the sequences identified in this study were compared with reference genotypes (A and E) from the NCBI database, all of which are genotype-matched references. The mutations observed are deviation from the standard or reference nucleotide sequence associated with that particular genotype, and its significance is often evaluated based on its impact on viral function, immune evasion, or resistance to antiviral therapies.

Figure 1:  the black background is distracting and should be removed.

In the revised version, the figure has been replaced as TIFF file with a white background.

Figure 2:  this should be redrawn as a phylogram such that branch lengths denote genetic distance.

Thank you for the suggestion. The phylogenetic tree has been redrawn (uploaded as Fig 2) with branch lengths now representing genetic distance.

How different are the various sequences at the nucleotide level?

At the nucleotide level, 298 out of 322 nucleotides (92.5%) are conserved across the 14 different HBV DNA-positive sequences.

What HBV genotypes have been found chronic HBV infections in Cameroon?

The HBV genotypes show different geographical distribution in populations around the globe. The most cosmopolitan genotypes are A and D, which are prevalent in Europe, Africa and North America. While Genotypes B and C are most common in East and Southeast Asia, and genotype E is mainly restricted to Africa. Genotype F is indigenous to the Americas and is found in native populations from Alaska, Central and South America.

This has been introduced in the discussion referenced as below,

*‘Previous studies have shown that genotypes A and E co-circulate in Cameroon, with reported mixed infections and recombination between genotypes A and E’.*

Does this region of S overlap with sites associated with drug resistance in P?  what drug resistance mutations were observed?

This had been included in the revised version in the discussion as below with approporiate references

*‘The primers targeted the overlapping S/P region of the HBV genome, spanning nucleotides 455 to 786, which includes the Reverse Transcriptase (RT) domain of the Polymerase gene—a region associated with resistance to nucleoside/nucleotide analogues (NAs). Upon analysis using geno2pheno and the HBV database, no well-characterized drug-resistance mutations were detected. However, some of the mutations we identified, such as rtW153R and rtI163V, have been associated with resistance to Adefovir and Lamivudine in previous studies investigating RT mutations in both treated and untreated individuals’*

**Reviewer #1:** This study is relevant to the transfusion field in Africa. However, some aspects need to be improved, especially the methodology.

Title
1.Is the sample sufficient to determine prevalence? I suggest changing the title.

We acknowledge that the sample size in our study is relatively modest, and identifying 14 cases of occult HBV infection is not unexpected, especially in Southwestern Cameroon, where the HBsAg positivity rate ranges from 8-15% (<https://pubmed.ncbi.nlm.nih.gov/28667212>, reference 2 in the draft).

The required sample size based on our power calculation was 225. Using available regional studies and our sample calculation (Sample size n = [DEFF*Np(1-p)]/ [(d2/Z21-α/2*(N-1)+p*(1-p)]), we estimated that investigating 225 blood donors (with 80% power and a 95% two-sided confidence interval) would allow us to detect the incidence in the general population. However, our current study included a larger sample size of 288 participants. This study is the first of its kind in this anglophone region of Cameroon, where the majority of the country is francophone. Additionally, Northern Cameroon has faced years of insurgency and violence from Boko Haram, highlighting the significance of research in this region to assess the broader implications of occult hepatitis B infection among blood donors.

From the title prevalence has been changed to incidence and is reflected now as

‘*Incidence of Occult Hepatitis B Infection (OBI) and hepatitis B Genotype Characterization Among Blood Donors in Cameroon’*

Abstract

2.It should be made clear in the abstract that these are HBsAg-negative blood donors.

Thank You. This has been amended in the revised abstract as below:

*‘Between March and June 2023, samples were collected from 288 HBsAg-negative blood donors aged 18 to 55 years and analysed for antibodies against the HBV core (anti-HBc) and surface antigens (anti-HBs).*’

Introduction
3.In the last paragraph, the authors mention the incidence of occult Hepatitis B. I suggest alignment with the title and taking into account comment 1.

The title has been modified from prevalence to incidence and now aligns with the statement in the last paragraph of the introduction.

Methodology
4. The recruiting process of 288 HBsAg negative blood donors needs to be clarified.
From which population were they recruited (blood donation candidates or approved donation candidates)? How many blood donors were included to obtain 288 HBsAg negatives? What was the frequency of HBsAg in these blood donors?

This had been clarified in the revised version as below

*‘Between March and June 2023, 288 consented, healthy blood donors aged 18 to 55 years were recruited from the Buea Regional Hospital blood bank in Cameroon. Demographic data, vaccination status, and blood donation history were recorded using a structured questionnaire. After meeting the eligibility criteria, which included being HBsAg-negative, having no medical history of chronic disease, and testing negative for HIV, HCV, and syphilis, 3 mL of blood was collected from each donor. The eligibility criteria for blood donation included being aged 18 to 65 years, weighing at least 50 kg, being in good general health, free from acute illness, with normal blood pressure and pulse rates, and having adequate hemoglobin levels as determined during pre-donation screening. Individuals with chronic diseases, those on medication, and pregnant women are not eligible to donate. The HBsAg status, HIV, HCV, and Syphilis was determined using rapid test kits and ELISA (Fortress Diagnostics, Antrim, United Kingdom).The HBsAg assay used a sensitivity of 99.75% and a specificity of 99.87%, while the anti-HCV assay had a sensitivity of 99.79% and a specificity of 99.55%. Additionally, the HIV (Ag/Ab) assay had an analytical sensitivity for p24 antigen of 5 pg/mL, and the Treponema pallidum antibody rapid test kit showed a sensitivity of 100% and a specificity of 99.7%.’*

Regarding the second part of your question, during the sampling period from March to June 2023, we identified four HBsAg-positive blood donations as per the blood bank results. After testing these four samples with nested and real-time PCR, all four HBsAg-positive samples were confirmed as negative. Consequently, we classified these as false positives for HbsAg. Thus no HBsAg positivity among blood donors were observed.

5. I suggest a general description of the criteria for blood donation mentioned in number 113.

This general description and criteria for blood donation has been included in the revised version.

6. Serological tests have a low sensitivity for HBV (HBsAg) screening compared to molecular testing. What strategies were used to reduce false negative results?

We acknowledge that serological tests have lower sensitivity for HBV (HBsAg) screening compared to molecular testing. Although, Nucleic acid testing will be appropriate, blood banks in Cameroon, including the Buea Regional Hospital where the donors were recruited, rely solely on serological tests for HBV screening. This is equally the message from this study *‘The results suggest a considerable risk of transfusion-transmitted HBV in this region. Therefore, to ensure blood safety, nucleic acid testing (NAT) is recommended, as relying solely on HBsAg assays is insufficient to eliminate this risk.’* which is reflected in the concluding statements.

7. I suggest that you also indicate the sensitivity and specificity of the tests used for other serological markers.

Thank you for the suggestion, and this is now included in the methodology section in the revised verison.

8.What is the detection limit of the quantitative test used for HBV.

The limit of detection for the real-time PCR used to quantify HBV viral load was 25 IU/mL. This limit was established using a control plasmid with a concentration of 10^6 copies/µL, serially diluted tenfold. The linear equation relating Ct values to log (copies/µL) was used to calculate viral loads. The highest Ct value detected, which was 40, corresponded to 25 IU/mL and was therefore considered the detection limit.

This information has been added in the methods section.

Results

9.Sample BD229 has a high viral load. Without confirmatory testing for HBsAg, we cannot exclude the possibility of it being a sample in an immunological window period or a false negative in the serological test.

We agree with you that this sample might have been a misdiagnosis, especially considering the high viral load of > 200IU/ml, which is typical for OBI. This was also mentioned in the last paragraph of the discussion section. In addition, we have also clarified that this is a ‘false’ OBI sample.

*‘In one sample with a high viral load (>2000 IU/mL), one mutation (sA184V) was observed which also occurred in nine other OBI sequences. Despite the high viremia, qualitative ELISA HBsAg assay used in the blood bank, which has a detection limit of 0.5 ng/mL, failed to detect it, suggesting a potential laboratory misdiagnosis’*

Discussion

10. Despite this being part of the limitation, it is not clear why HBsAg-positive samples were excluded.

We acknowledge that excluding HBsAg-positive samples is a limitation of this study, especially given that our focus was on approved blood donors who were presumed to be free of HBV infection. Our investigation concentrated on HBsAg-negative donors to evaluate and highlight the potential risk of HBV transmission in this population, despite existing pre-donation screening procedures.

**Reviewer #2:**

This study investigated the prevalence of OBI, which is defined by the presence of serum viral DNA for hepatitis B, associated viral factors, and the HBV genotype circulating in blood donors in southwestern Cameroon who tested negative for HBsAg and concluded that relying on routine HBsAg testing is insufficient to prevent HBV transmission through transfusions in endemic regions and recommend supplementing HBsAg testing with nucleic acid amplification testing (NAT) and/or anti-HBc testing as surrogates for detecting OBI.

Abstract
• The abstract mentions only anti-HBs and anti-HBc testing. However, the HBsAg test was not included and was only mentioned in the methodology section. HBsAg tests should also be included.

We thank the reviewer for raising this constructive suggestion. This has been clarified in the abstract and methodology section.

*‘Between March and June 2023, samples were collected from 288 HBsAg-negative blood donors aged 18 to 55 years and analysed for antibodies against the HBV core (anti-HBc) and surface antigens (anti-HBs).’*

Methods

• The researcher should describe the design of the study. Is this a cross-sectional study? If so, this needs to be explained

• The researcher should describe the study design. Is it a cross-sectional study? If so, it is necessary to explain this.

The revised version gives an detailed overview as below

*‘Between March and June 2023, 288 consented, healthy blood donors aged 18 to 55 years were recruited from the Buea Regional Hospital blood bank in Cameroon. Demographic data, vaccination status, and blood donation history were recorded using a structured questionnaire in this cross-sectional study. After meeting the eligibility criteria, which included being HBsAg-negative, having no medical history of chronic disease, and testing negative for HIV, HCV, and syphilis, 3 mL of blood was collected from each donor. The eligibility criteria for blood donation included being aged 18 to 65 years, weighing at least 50 kg, being in good general health, free from acute illness, with normal blood pressure and pulse rates, and having adequate hemoglobin levels as determined during pre-donation screening. Individuals with chronic diseases, those on medication, and pregnant women are not eligible to donate. The HBsAg status, HIV, HCV, and Syphilis was determined using rapid test kits and ELISA (Fortress Diagnostics, Antrim, United Kingdom).The HBsAg assay used a sensitivity of 99.75% and a specificity of 99.87%, while the anti-HCV assay had a sensitivity of 99.79% and a specificity of 99.55%. Additionally, the HIV (Ag/Ab) assay had an analytical sensitivity for p24 antigen of 5 pg/mL, and the Treponema pallidum antibody rapid test kit showed a sensitivity of 100% and a specificity of 99.7%.’*

• Line 122: What is the titre value of the positive anti-HBc test? Give the value of the positive anti-HBc test, as the researcher have done for anti-HBc.

The anti-HBs test is a quantitative assay for which the titre values are provided. The anti-HBc assay is a qualitative assay where the presence or absence of anti-HBc antibodies was determined by comparing for each sample the recorded absorbances with that of the calculated cut-off value (mean of absorbance values for the positive control serum divided by 5), following manuafcturers instructions.

This has been added in the methodological section as below

*‘The anti-HBs positivity was defined as a titer value >10 mIU/mL and the assay had a sensitivity of 99.2% and a specificity of 99.4%. The anti-HBc assay (qualitative) had a sensitivity of 99.53% and a specificity of 99.9%.’*

• Be consistent with the writing of the markers in some the researcher used both higher case letter and lower-case letters and it may be confusing to the reader e.g. Anti-HBs/ anti-HBs.

We thank the reviewer for this valuable comment. We have amended this all through the manuscript as ‘anti-HBs and anti-HBc’.

• Briefly describe the method (statistical analysis and calculations) used to calculate the prevalence, median age and viral load as the age in median, viral load in mean/ average were reported.

The revised version gives an detailed overview as below

*‘Statistical analysis was performed using R version 4.0. The median age of the blood donors was determined as the median value in the ordered age distribution, with variability described as interquartile range (IQR). The incidence of OBI was calculated as the proportion of HBsAg-negative blood donors who tested positive for HBV DNA and with a viral load not exceeding 200 IU/mL. Viral load in positive samples was quantified using real-time PCR, with results expressed in IU/mL based on a standard curve generated from a serially diluted control plasmid with a concentration of 10^6 copies/µL.’*

Results
• The HBsAg results are not reported, it is not clear if the researcher performed the HBsAg test, or it was previously tested prior to the study. If it was performed, this should be described as well.

Thank you for raising this question. The HBsAg test was conducted by the blood bank as part of the routine procedure for selecting blood donors. We subsequently performed serological testing for other markers (anti-HBs and anti-HBc) and NAT. This has also been clarified in the methods section.

• Lines 170 – 173: Anti-HBs and anti-HBc are reported twice. The anti-HBs as 29% and 3%; and anti-HBc as 58% and 32%.

This has been amended in the revised version as below

*‘The serology results for HBV showed that 58% (n=167/288) were positive for anti-HBc and 29% (n=83/288) positive for anti-HBs. Of these, 26% (n = 75/288) tested positive for both antibodies and 39% (n=113) tested negative for both anti-HBc and anti-HBs antibodies (Fig 1).’*

• The results section needs to be revised. For example, 26% (n = 75) tested positive for both antibodies and 39% (n=113) were negative for both markers. When one subtracts 75 from 288 The researcher gets 213 and not 113. What is the status of the 100 samples. I suggest that the researcher revise these results.

We appreciate the reviewer’s insightful comment and suggestion. The remaining 100 samples are those that were positive for only one of the two markers as shown in Fig 1, i.e., anti-HBc (+) anti-HBs (-) = 92, and anti-HBc (-) anti-HBs (+) = 8 adding up the 100 samples. However, we agree that this section needs revision for better understanding. We have resolved to present the general positivity rates (i.e. 58% (n=167/288) positivity for anti-HBc, and 29% (n=83/288) positivity for anti-HBs), co-positivity (i.e. 26% (n = 75/288) positivity for both antibodies), and negativity rates for both markers ( i.e. 39% (n=113) negative for both antibodies) and the details of sole positivity rates can be found in Fig 1.

• Line 184: Almost all tested positive remained negative for anti-HBs (13/14; 93%).
• Since the researcher reports the prevalence, the word “almost” should not be used and the researcher should provide the frequency in numbers or % only.

This statement has been revised accordingly as

*‘Of all samples tested positive for HBV DNA, 93% remained negative for anti-HBs (Table 1).’*

• Among these samples, four had detectable viral loads measured by qPCR, ranging from <10 to 2593 IU/mL. The results report on the viral load, but not on the OBI. Mention how many samples are OBI. Also, clarify if the sample (BD299) with a viral load of 2593 IU/mL considered OBI. According to the OBI definition, the viral load is <200 IU/mL.

We appreciate for this comment. This has been clarified in the results section as:

*‘HBV DNA was detected in 14 out of 288 samples (5%) by nested PCR. The median age of the HBV DNA-positive blood donors was 24 years (range: 18-41). Among these samples, four had detectable viral loads measured by qPCR, ranging from <25 to 2593 IU/mL (Table 1). Three of those with detectable viremia were positive for anti-HBc, while one was positive for anti-HBs but not anti-HBc. Of all samples tested positive for HBV DNA, 93% remained negative for anti-HBs (Table 1). Following the consensus definition of OBI, one HBV DNA positive sample (BD229) was considered ‘false’ OBI since its viral load is > 200IU/mL. Therefore, the incidence of OBI in this population is 4.5%.’*

Phylogenetic tree

• Be consistent with writing figure 2 or Fig 2.

All inconsistencies have been corrected. Thank you.

• The reference sequences are not written in the correct standard order, e.g., the correct way of writing ref. seq is genotype accession no|location. The description of the identity of the study sequences with the reference sequences is not clear, since the reference sequences are not written correctly.

We thank the reviewer for this comment. The reference sequences used to reconstruct the phylogenetic tree have been rewritten as accession number|genotype location, as shown on the newly reconstructed tree in the revised version.

• Under methods section, it is mentioned that the bootstrap of 1000 replicates was used to create the tree and that the nodes indicate indicated the bootstrap values. the bootstrap values are not included in the phylogenetic tree, so this tree does not represent a bootstrap phylogenetic tree. See lines 213-214.

We agree that the bootstrap values were not visible on the tree as stated in the text because of the black background color of the uploaded image. The bootstrap values are now visible on the newly reconstructed phylogenetic tree.

• Phylogenetic analysis with 30 reference sequences corroborated these findings (Fig 2). The image has poor quality and resolution. I suggest that the researcher revise the construction of the phylogenetic tree.

Thank you for your suggestion, the construction of the phylogenetic tree has been revised and presented on a white background. If the image quality is still poor, it might be due to the system automatically reducing the resolution and we kindly ask the reviewer that the respective figure can be downloaded as a high resolution image in the right corner of the pdf file.

• Since reference genomes (U87742 for genotype A and AB091256 for genotype E) were used for constructing a phylogenetic tree. Is there any reason why they are excluded for mutation analysis comparison?

Thanks for rasing this question. The sequences used for the mutation analysis were two of the sequences (one each for genotypes A and E), which is equally used for the phylogenetic tree reconstruction.

This might be overlooked and been missed, as we used the genome accession number of the sequences for the tree whereas the protein_ID (Surface and RT domain of Polymerase gene) of the same genome sequence for the mutation analysis. This has been corrected by using only the genome accession number of the reference sequences for the tree reconstruction and also for the mutation analysis comparison.

• Under S1 and S2- write the genotypes of the reference sequences AAC58023.3, BAC65109.1, AAM12945.1, BAC65108. Why did the researcher use different reference sequences for the S and RT regions? Combine the standard reference sequences in both alignments for the S and RT regions so that we know the genotypes from which these OBI mutations have been previously identified.

Thank you for your suggestion. The alignment of the S and RT regions has been revised to combine the reference sequences of the identified genotypes (A and E). Also, the genome accession numbers|genotype of the reference sequences have been added to the alignment which are the same references used to reconstruct the phylogenetic tree. The new alignments have been uploaded again as S1 Fig for the S region and S2 Fig for the RT region.

• The reference sequence on the top ‘AAC58023.3’ has a mutation 131N like the samples, which indicates it might not be a wild-type sequence because the other reference sequence ‘BAC65109.1’ below has wildtype base T131. Similarly, AAC58023.3 has T143 and “BAC65109.1 has S143.

We appreciate your comment. The initial alignment was presented separately for each identified genotype (genotype A above; one sequence only, and genotype E below; 13 sequences). In the major hydrophilic region of the HBsAg, positions 131 and 143 are variable positions and could be occupied by amino acids N/T (for position 131) and S/T (for position 143). Genotype A sequences have 131N while genotype E sequences have 143S (https://pubmed.ncbi.nlm.nih.gov/27053845/). For better understanding, we have also shown this on the amino acid alignment below for 20 sequences (10 for each genotype) randomly selected from the HBV database. Further analysis of these sequences using geno2pheno (<https://hbv.geno2pheno.org/index.php>) for mutations, identified T131N and S143L as HBsAg escaped mutations as shown in the screenshots below.

U87742|A

M57663|A

X02763|A

EU054331|A

AB030513|A

EU859945|A

FJ692562|A

AB194950|A

AF090842|A

X51970|A

AB106564|E

X75657|E

AB032431|E

AB091257|E

AB194947|E

DQ060822|E

GQ161824|E

HM363609|E

KU736897|E

LT623861|E

• Is there a reason why Y206C is not reported on the results, as it shows to be a variant in the alignment (S1)

Thank you for pointing our attention to this missed variant. This omission was unintentional. The variant Y206C has been included in the results section and its significance discussed.

• Is there a reason for the separate alignment of the reference sequence ‘AAM12945.1’ and sample BD14 on top and with the other sequences below? I suggest The researcher combine the alignments and have all the alignment in one image/page. I suggest that the researcher include 2 or more standard references for both genotypes A and E.

Dear reviewer, the alignment was genotype-matched and so separated into genotype A and E sequences and references. As suggested, two reference sequences per genotype have been used for the combined new alignment, and images uploaded as S1 Fig and S2 Fig.

Discussion

• In one sample with a high viral load (>2000 IU/mL), two mutations (sA184V and rtH125Q) 280 were observed that also occurred in other OBI sequences. On the basis of this statement above, is this sample having OBI or not. The load is high for it to be reported as OBI. The detection limit of 0.5 282 ng/mL is quite low to fail in the detection of HBsAg suggesting that the was nothing wrong with the HBsAg testing. The same sample with a high viral load has mutations that are different from the other samples, and the researcher did not discuss these differences and what could have impacted on this type of results.

We appreciate the reviewer for this valuable comment. We realized that the reference sequence used before for the alignment for genotype E sequences was not a wild-type sequence and so 100% of genotype E sequences presented with the mutation rtH125Q. This has been fixed in the new alignment uploaded using two standard reference sequences (from the HBV genotyping tool) and this rtH125Q mutation wasn’t observed in any of the sequences as 125Q is present in the references. This has been revised in the manuscript as well.

The one sample with a high viral load now presents with only one mutation also present in nine other samples. Given the high viral load for this sample, we agree that this must have been a misdiagnosis. This is discussed in the last paragraph of the discussion section and has been clarified in the results section.

• The researcher identified the mutations associated with drug resistance in the RT region; however, the researcher did not discuss how they impact on the OBI or the overlapping S region. This highlights another limitation of the study, which is the lack of information on the treatment history of the patients.

Thank you. To clarify, the investigations were conducted on healthy blood donors who were HBsAg-negative but were found to carry HBV DNA in low copy numbers. When we characterized their HBV genotypes and mutations, we identified specific mutations in these occult HBV infection (OBI) individuals. None of these individuals had a history of ongoing or previous treatment.

The mutations observed, such as rtW153R and rtI163V, have been associated with resistance to Adefovir and Lamivudine, as reported in previous studies investigating these RT mutations in both treated and untreated individuals (<https://pubmed.ncbi.nlm.nih.gov/23026293/>, <https://pubmed.ncbi.nlm.nih.gov/37262110/>). However, the identification of these mutations alone does not allow us to predict their potential impact if an OBI individual were to progress to a full-blown acute hepatitis B infection. This has been clarified in the discussion section. Also, we have discussed in general the impact of RT mutations on OBI and the overlapping S region.

• The significance of the study results is not well discussed; the researcher needs to discuss the clinical implications of the OBI prevalence and mutations either towards e.g. diagnosis or treatment.

We sincerely thank you for your insightful comments and suggestions, which greatly improved the quality of our manuscript. The clinical impact of OBI prevalence cannot be imputed here, as the study only investigated OBI incidence, and if so, which of these HBV genotypes are circulating in OBI individuals. We investigated whether these variations in the HBV genome are specific for OBI. However, none of the variants is clearly associated with OBI. Although other non-synonymous SNPs were also observed in this study, they have been shown to be associated with the therapeutic response. We only report the observation of such SNPs in this OBI group carrying different genotypes and cannot infer any possible association with treatment response from these results.

In response to your comments, we have strengthened the discussion section to better highlight these aspects. We hope that these revisions more effectively convey the intended message of our research.
